# Development of a Conceptual Implant Stability Index Framework for Computational Risk Assessment in Implant Dentistry

**DOI:** 10.3390/bioengineering13060684

**Published:** 2026-06-12

**Authors:** Liliana Sachelarie, Corina Laura Ștefănescu, Rodica Maria Murineanu, Mircea Grigorian, Agripina Zaharia, Loredana Liliana Hurjui

**Affiliations:** 1 Department of Dental Medicine, Apollonia University, 700511 Iasi, Romania; 2 Faculty of Dentistry, Ovidius University, 900527 Constanta, Romania; rodica.murineanu@365.univ-ovidius.ro (R.M.M.); mircea.grigorian@365.univ-ovidius.ro (M.G.); 3 Private Dental Practice, 900162 Constanta, Romania; agrizaharia@yahoo.com; 4 Faculty of Medicine, Grigore T. Popa University of Medicine and Pharmacy, University Street 16, 700115 Iasi, Romania; loredana.hurjui@umfiasi.ro

**Keywords:** dental implant instability, Implant Stability Index, computational bioengineering, conceptual computational model

## Abstract

**(1) Background**: Dental implant stability is influenced by multiple biomechanical, implant-related, and systemic factors, including bone density, implant geometry, biomechanical loading, smoking, osteoporosis, and diabetes mellitus. Computational bioengineering approaches may facilitate theoretical assessment of implant stability and support future risk-evaluation strategies. The aim of this study was to develop a conceptual computational framework for assessing theoretical implant instability using clinically relevant biomechanical and systemic parameters. **(2) Methods**: A multivariable computational framework was developed by integrating bone density, implant dimensions, implant mobility indicators, biomechanical loading conditions, smoking status, osteoporosis, and diabetes mellitus into a conceptual Implant Stability Index (ISI). Computational simulations and theoretical risk stratification procedures were used to evaluate framework behavior under different simulated conditions. **(3) Results**: The framework demonstrated the theoretical ability to differentiate between favorable and unfavorable implant stability conditions. Reduced bone density, increased implant mobility indicators, excessive biomechanical loading, and adverse systemic factors resulted in lower calculated ISI values and a higher theoretical instability risk. The framework further enabled the classification of simulated conditions into high-, moderate-, and increased-instability-risk categories. **(4) Conclusions**: The proposed Implant Stability Index represents a conceptual computational framework for integrating biomechanical, implant-related, and systemic factors associated with implant stability. Although not clinically validated, the framework may provide a proof-of-concept foundation for future studies involving clinical datasets, biomechanical simulations, and advanced computational modeling approaches.

## 1. Introduction

Dental implants have become one of the most predictable and widely used therapeutic options in modern oral rehabilitation, significantly improving oral function, esthetics, and quality of life in patients with partial or complete edentulism [1]. Despite the high success rates reported over recent decades, implant instability and implant failure remain important clinical concerns that may compromise long-term treatment outcomes [2]. Implant instability is a multifactorial phenomenon influenced by biomechanical, biological, systemic, and local clinical parameters, including bone quality, peri-implant stress distribution, implant geometry, occlusal loading, smoking, osteoporosis, diabetes mellitus, and peri-implant inflammatory conditions [3,4].

Implant stability is generally classified into primary stability and secondary stability. Primary stability is mainly mechanical and depends on bone density, implant design, and surgical insertion conditions, whereas secondary stability is biologically achieved through osseointegration and peri-implant bone remodeling [5]. The disruption of these processes may lead to implant micromobility, marginal bone loss, impaired osseointegration, and ultimately implant failure [6]. Consequently, the accurate assessment and early prediction of implant instability represent essential objectives in implant dentistry.

Several diagnostic and monitoring methods have been proposed to evaluate implant stability, including Resonance Frequency Analysis (RFA), Periotest measurements, insertion torque evaluation, radiographic analysis, and finite element biomechanical assessment [7,8]. Among these approaches, RFA has become widely accepted for its non-invasive nature and its ability to provide quantitative Implant Stability Quotient (ISQ) values for longitudinal implant monitoring [9]. Similarly, Periotest analysis allows the evaluation of implant mobility and peri-implant damping characteristics [9,10]. However, these methods are often interpreted in isolation and may not fully capture the complex interactions among biomechanical, systemic, and local biological factors that influence implant stability.

Recent advances in computational bioengineering, conceptual analytics, and digital dentistry have created new opportunities to develop conceptual computational models that integrate multiple clinical and biomechanical variables into objective risk assessment systems [11]. Previous studies have explored implant stability using a variety of approaches, including Periotest measurements, resonance frequency analysis, finite element modeling, and biomechanical simulations [9,12]. More recently, artificial intelligence and machine-learning-based prediction systems have emerged as promising tools for implant planning, risk assessment, and outcome prediction in implant dentistry [12]. These methodologies have substantially improved the understanding of implant stability behavior and risk assessment. However, many of these approaches focus primarily on specific biomechanical measurements, simulation environments, or data-driven prediction strategies. The present study does not aim to replace these approaches but rather to propose a conceptual computational framework that integrates routinely available biomechanical, implant-related, and systemic variables into a unified Implant Stability Index (ISI) for assessing theoretical stability.

Such computational and data-driven approaches have contributed substantially to the advancement of implant stability assessment and personalized treatment planning. Nevertheless, controversy persists regarding the relative contributions of systemic diseases, bone quality, mechanical overload, and peri-implant biomechanical stress to implant instability and failure [12]. In addition, many currently available computational and conceptual methodologies primarily focus on specific biomechanical measurements, simulation environments, or data-driven algorithms, which may limit the integration of routinely available clinical parameters into a unified assessment framework. In contrast, the proposed Implant Stability Index (ISI) was developed as a conceptual computational framework that integrates systemic, biomechanical, and implant-related variables into a single theoretical model of stability assessment. The framework was designed to explore the potential interaction of these factors within a unified computational structure rather than to provide a clinically validated conceptual tool.

The developed framework was designed as a multivariable computational integration system that combines biomechanical, implant-related, and systemic parameters into a conceptual scoring system for assessing theoretical implant stability.

The aim of the present study was to develop a conceptual computational framework for the theoretical assessment of dental implant instability by integrating clinically relevant biomechanical and systemic parameters associated with implant stability. The study further aimed to develop a computational risk assessment framework to estimate implant instability risk and stratify theoretical clinical scenarios into low-, moderate-, and high-risk categories. The developed framework may provide a conceptual basis for future investigations exploring computational approaches to implant stability assessment and methodological development in implant dentistry.

## 2. Materials and Methods

### 2.1. Computational Model Design

A conceptual computational framework for dental implant instability was developed based on biomechanical and clinical parameters commonly associated with implant stability in the current scientific literature. The model was designed to simulate the multifactorial interactions that influence implant prognosis and to provide a theoretical computational approach to estimating implant stability in oral rehabilitation.

The ISI computational stability framework was conceived as an integrated risk architecture designed to simulate systemic-biomechanical interactions associated with implant stability behavior under different theoretical clinical conditions.

The computational framework integrated variables recognized as relevant to implant stability assessment, including bone density, implant dimensions, implant location, biomechanical loading conditions, implant mobility indicators, smoking status, osteoporosis, and diabetes mellitus, based on factors previously associated with implant stability and survival in the literature [4,7,8]. These parameters were selected due to their reported influence on osseointegration, peri-implant biomechanical stress distribution, and implant survival.

The framework was conceived as a mathematical model for implant stability verification that integrates routinely available implant dentistry parameters into a theoretical computational framework. The model was developed to estimate theoretical implant stability behavior under different biomechanical and systemic conditions.

### 2.2. Selection of Biomechanical and Clinical Parameters

The conceptual variables included in the computational model were selected following a comprehensive review of the current literature regarding factors associated with implant instability and implant failure [4,7,13]. The analyzed parameters were grouped into systemic, biomechanical, and implant-related categories.

Systemic variables included smoking status, osteoporosis, and diabetes mellitus due to their documented effects on bone metabolism, healing capacity, and osseointegration [5,6,7]. Biomechanical variables included bone density, loading conditions, and implant mobility assessment parameters commonly associated with peri-implant micromobility and stress behavior [1,8,9,10]. Implant-related variables included implant dimensions and implant location, as these factors may influence stress distribution and mechanical stability under functional loading conditions.

Each parameter was incorporated into the computational framework based on its potential contribution to implant stability, as reported in previous biomechanical and clinical studies.

### 2.3. Mathematical Implant Stability Model

A mathematical implant stability model was developed to estimate theoretical implant stability under different biomechanical and systemic conditions. The developed model integrated the selected variables into the following Implant Stability Index (ISI):

$$ISI=\frac{B\times D_{i}\times L_{i}}{PTV\times LF\times SF}$$

where ISI = Implant Stability Index; B = Bone density factor; $D_{i}$ = Implant diameter factor; $L_{i}$ = Implant length factor; PTV = Periotest value; LF = Loading Factor; SF = Systemic Factor.

The proposed Implant Stability Index (ISI) was developed as a parameter-interaction model that integrates biomechanical, implant-related, and systemic variables into a unified computational assessment framework. The proposed framework was conceptually inspired by previously reported computational, finite element, and biomechanical approaches used to investigate implant stability and peri-implant stress behavior [11,12,13].

Parameters theoretically associated with improved implant stability were positioned in the numerator, whereas variables associated with increased instability risk were incorporated into the denominator. The multiplicative structure of the proposed equation was intentionally selected as a simplified conceptual approach to represent the cumulative influence of biomechanical, implant-related, and systemic parameters on theoretical implant stability behavior. The equation was developed as a proof-of-concept computational integration framework and was not intended to represent a clinically calibrated regression model or a validated conceptual algorithm.

**Figure 1:**
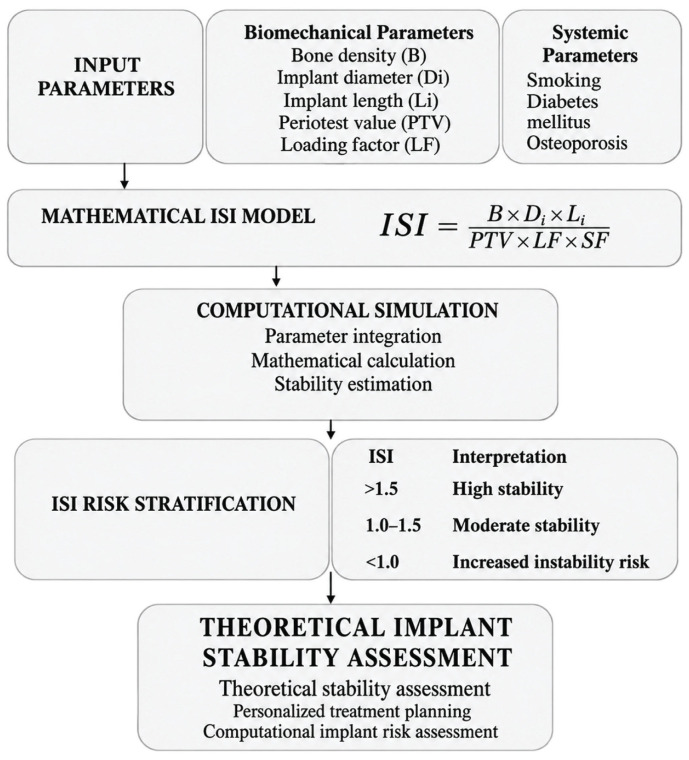
Computational workflow of the developed Implant Stability Index (ISI) model integrating biomechanical, implant-related, and systemic parameters for theoretical implant stability prediction and computational risk stratification.

Within the developed framework (Figure 1), higher ISI values were theoretically associated with improved implant stability, whereas lower ISI values indicated increased theoretical risk of implant instability.

### 2.4. Computational Parameter Classification and Risk Stratification

To simulate different theoretical clinical conditions, biomechanical and systemic parameters were classified into predefined computational categories. Normalized theoretical parameter values were assigned in order to simulate the computational behavior of the developed Implant Stability Index under different biomechanical and systemic conditions.

The selected variables were derived from clinically recognized factors known to influence implant stability and osseointegration, including bone quality, implant geometry, loading conditions, implant mobility indicators, and systemic health factors. To enable computational integration within the conceptual framework, these variables were transformed into normalized theoretical coefficients representing favorable, intermediate, and unfavorable conditions. The assigned coefficients were intended as illustrative theoretical values designed to simulate relative differences among clinical scenarios and should not be interpreted as clinically validated weighting factors, Table 1.

**Table 1:** Normalized theoretical parameter values used in the computational simulation model.

Parameter	Low	Moderate	High
Bone density factor (B)	1.0	1.5	2.0
Implant diameter factor (Di)	1.0	1.2	1.4
Implant length factor (Li)	1.0	1.2	1.4
Periotest value factor (PTV)	1	3	5
Loading factor (LF)	1.0	1.5	2.0
Systemic factor (SF)	1.0	1.3	1.6

Loading conditions were categorized according to the theoretical intensity of biomechanical stress applied to the implant structure, Table 2:

**Table 2:** Classification of biomechanical loading conditions and Loading Factor (LF) values.

Loading Condition	LF
Favorable loading	1.0
Moderate loading	1.5
High biomechanical loading	2.0
Severe overload/bruxism	2.5

Systemic conditions were incorporated into the model using a theoretical systemic factor associated with impaired bone remodeling and osseointegration, Table 3:

**Table 3:** Systemic condition classification and Systemic Factor (SF) values.

Systemic Condition	SF
No systemic condition	1.0
Smoking	1.2
Diabetes mellitus	1.4
Osteoporosis	1.5
Multiple systemic risk factors	1.8

Based on the calculated ISI values, a computational risk stratification framework was developed to classify theoretical implant stability into three conceptual categories, Table 4:

**Table 4:** Computational Implant Stability Index (ISI) risk stratification categories.

ISI Value	Theoretical Stability Category
>1.5	High stability
1.0–1.5	Moderate stability
<1.0	Increased instability risk

The stratification framework was intended to simulate potential clinical applications of the developed computational model in implant dentistry and oral rehabilitation.

### 2.5. Computational Simulation and Statistical Analysis

Normalized theoretical parameter values were used to simulate the computational behavior of the developed Implant Stability Index under different biomechanical conditions.

Computational simulations were performed to evaluate the theoretical behavior of the developed Implant Stability Index under different biomechanical and systemic conditions [11,13]. Multiple simulated scenarios combining different values of bone density, Periotest measurements, implant dimensions, loading conditions, and systemic factors were analyzed to assess their influence on theoretical implant stability.

Descriptive computational analyses and simulation-based parameter interaction assessments were used to evaluate the relative influence of the selected variables on calculated implant stability behavior. The computational consistency of the model was evaluated under various simulated loading and systemic conditions to assess the theoretical robustness and applicability of the developed framework.

## 3. Computational Simulation Results

The presented computational simulations were designed to demonstrate the internal behavior and mathematical consistency of the proposed conceptual Implant Stability Index (ISI) framework. The objective of these analyses was not to provide clinical validation, conceptual performance metrics, or independently derived clinical observations, but rather to examine how the framework behaves under different theoretical combinations of biomechanical, implant-related, and systemic parameters. The generated trends were intended to illustrate the behavior of the proposed framework and to assess whether the simulated outcomes remained consistent with established observations reported in the implant dentistry literature.

### 3.1. Mathematical Behavior of the Implant Stability Model

The computational simulations enabled the theoretical evaluation of the behavior of the proposed Implant Stability Index (ISI) framework under different combinations of biomechanical, implant-related, and systemic conditions. Within the framework, increased bone density and favorable implant dimensions generated higher calculated ISI values, reflecting the mathematical structure of the model in which these parameters contribute positively to the theoretical stability assessment.

The computational framework illustrated how variations in biomechanical and systemic parameters influenced the calculated ISI values under simulated conditions. In particular, unfavorable implant mobility indicators, excessive biomechanical loading conditions, and adverse systemic factors generated progressively lower ISI values within the framework. Similarly, simulations incorporating multiple unfavorable conditions produced lower theoretical stability profiles than simulations characterized by predominantly favorable parameters.

The generated trends were intended to illustrate the internal behavior of the proposed conceptual framework and to evaluate whether the simulated outcomes remained consistent with established observations reported in the implant dentistry literature. Consequently, the presented findings should be interpreted as illustrative computational simulations rather than validation outcomes or measures of conceptual performance.

### 3.2. Influence of Implant Mobility Parameters on Computational ISI Values

The computational simulations generated progressively lower calculated Implant Stability Index (ISI) values under theoretical conditions characterized by increased implant mobility indicator values (PTV), Table 5. Within the developed conceptual computational framework, elevated mobility indicators combined with unfavorable biomechanical and systemic conditions generated lower theoretical stability profiles and reduced calculated ISI values.

**Table 5:** Influence of implant mobility indicators on calculated Implant Stability Index (ISI) values.

Mobility Indicator Value (PTV)	Calculated ISI
1	2.1
3	1.7
5	1.2
7	0.8

The simulated computational analyses illustrated that lower implant mobility indicator values generated more favorable theoretical stability profiles within the framework, whereas progressively increased mobility-related parameters produced lower calculated ISI values. These trends are consistent with the established clinical interpretation of implant mobility as a factor associated with reduced implant stability.

The multivariable computational framework further illustrated how implant mobility parameters interact with biomechanical and systemic conditions within the conceptual model. Simulations incorporating elevated mobility indicators together with unfavorable biomechanical loading conditions and systemic modifying factors generated lower theoretical stability profiles than simulations characterized by predominantly favorable conditions. These findings should be interpreted as illustrative computational simulations demonstrating framework behavior rather than validation outcomes or measures of conceptual performance.

### 3.3. Influence of Bone Density on Theoretical Implant Stability

The computational simulations generated progressively higher calculated Implant Stability Index (ISI) values under theoretical conditions characterized by increased bone density factors, Table 6. This behavior reflects the mathematical structure of the proposed conceptual framework, in which bone density contributes positively to the calculated ISI values.

**Table 6:** Influence of bone density factor on the calculated Implant Stability Index (ISI).

Bone Density Factor	Calculated ISI
1.0	0.9
1.5	1.3
2.0	1.8

The simulated computational analyses illustrated that lower bone density factors generated less favorable theoretical stability profiles within the framework, whereas increased bone density factors produced higher calculated ISI values. These trends are consistent with established evidence indicating that favorable bone quality contributes positively to implant stability and osseointegration.

The computational framework further illustrated how variations in bone density may influence the model’s calculated stability profiles under different biomechanical and systemic conditions. The generated trends should be interpreted as illustrative computational simulations demonstrating framework behavior rather than validation outcomes or measures of conceptual performance.

### 3.4. Influence of Biomechanical Loading Conditions

The computational simulations generated progressively lower calculated Implant Stability Index (ISI) values under theoretical conditions characterized by increased biomechanical loading factors, Table 7. This behavior reflects the mathematical structure of the proposed conceptual framework, in which loading conditions contribute inversely to the calculated ISI values.

**Table 7:** Influence of biomechanical loading conditions on the calculated Implant Stability Index (ISI).

Loading Condition	LF	Calculated ISI
Favorable loading	1.0	2.0
Moderate loading	1.5	1.4
High biomechanical loading	2.0	0.9

The computational framework further illustrated how loading conditions interact with additional biomechanical and systemic parameters within the conceptual model. Simulations incorporating elevated loading factors together with reduced bone density or unfavorable systemic conditions generated lower theoretical stability profiles than simulations characterized by predominantly favorable conditions. These findings should be interpreted as illustrative computational simulations demonstrating framework behavior rather than validation outcomes or measures of conceptual performance.

### 3.5. Influence of Systemic Factors on Implant Stability

The computational simulations generated progressively lower calculated Implant Stability Index (ISI) values under theoretical conditions characterized by increasing systemic factor values, Table 8. This behavior reflects the mathematical structure of the proposed conceptual framework, in which systemic modifying factors contribute inversely to the calculated ISI values.

**Table 8:** Influence of systemic conditions on the calculated Implant Stability Index (ISI).

Systemic Condition	SF	Calculated ISI
No systemic condition	1.0	2.02
Smoking	1.2	1.68
Diabetes mellitus	1.4	1.24
Osteoporosis	1.5	1.05

The calculated computational behavior suggested that systemic conditions associated with impaired osseointegration and altered bone metabolism may negatively affect calculated implant stability profiles.

### 3.6. Computational Risk Stratification

Based on the calculated Implant Stability Index values, the developed computational framework enabled theoretical classification of implant stability into three conceptual categories, Table 9.

**Table 9:** Computational risk stratification based on Implant Stability Index (ISI) values.

ISI Value	Calculated Implant Stability
>1.5	High stability
1.0–1.5	Moderate stability
<1.0	Increased instability risk

The computational stratification model suggested that the simultaneous presence of unfavourable biomechanical and systemic parameters may substantially increase the theoretical risk of implant instability within the conceptual computational framework.

### 3.7. Simulated Computational Conditions

Several theoretical computational simulations were performed to evaluate the behavior of the developed conceptual Implant Stability Index (ISI) framework under different biomechanical and systemic conditions, Table 10. The generated outputs should be interpreted as illustrative computational simulations demonstrating framework behavior and internal mathematical consistency rather than clinical validation outcomes or measures of conceptual performance.

**Table 10:** Simulated computational conditions for theoretical implant stability prediction.

Computational Condition	Bone Density	Periotest Value	Loading Condition	Systemic Factor	Calculated ISI	Theoretical Stability Profile
Favorable stability profile	Favorable	Low PTV	Favorable loading	None	2.1	High stability
Moderate stability profile	Moderate	Moderate PTV	Moderate loading	Smoking	1.3	Moderate stability
Increased instability profile	Reduced	High PTV	High biomechanical loading	Osteoporosis + Diabetes	0.8	Increased instability risk

These demonstrated the influence of combined biomechanical and systemic parameters on calculated implant stability behavior. Favorable biomechanical conditions and the absence of systemic modifying factors were associated with elevated ISI values and improved theoretical implant stability profiles.

Conversely, unfavorable implant mobility indicators, reduced bone density, excessive biomechanical loading conditions, and adverse systemic modifying factors contributed to progressive reductions in calculated Implant Stability Index (ISI) values and increased theoretical implant instability risk within the computational model.

**Figure 2:**
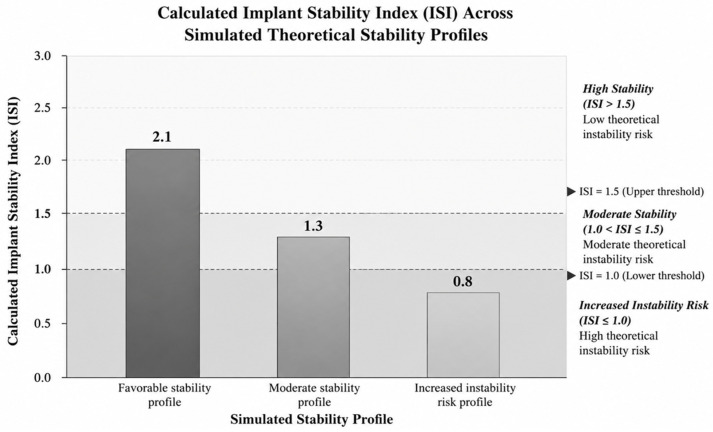
Comparison of calculated Implant Stability Index (ISI) values across simulated theoretical stability profiles.

Figure 2 illustrates the computational risk stratification generated by the proposed Implant Stability Index (ISI) framework across the simulated stability profiles. Favorable biomechanical and systemic conditions generated the highest calculated ISI values, whereas unfavorable conditions produced progressively lower stability profiles and increased theoretical implant instability risk.

## 4. Discussion

The present study proposed a conceptual computational framework for theoretical dental implant instability assessment by integrating biomechanical, implant-related, and systemic parameters into a unified Implant Stability Index (ISI). The developed model was designed to simulate the complex interactions that may influence implant stability behavior and peri-implant biomechanical adaptation under different theoretical clinical conditions.

The present computational framework was not designed to evaluate Periotest-derived implant mobility in isolation, but rather to integrate multiple interacting biomechanical, implant-related, and systemic variables into a unified computational Implant Stability Index (ISI).

Dental implant stability remains one of the principal determinants of long-term implant success and the quality of osseointegration [13]. Previous studies have demonstrated that implant instability is influenced by multiple interacting factors, including bone quality, implant geometry, occlusal loading, smoking, osteoporosis, diabetes mellitus, and peri-implant biomechanical stress distribution [4,5,14,15]. However, most currently available assessment methods evaluate these parameters individually and may not fully integrate their cumulative biomechanical effects into a unified conceptual system [16].

The present study sought to address this limitation by integrating multiple clinically relevant biomechanical, implant-related, and systemic variables into a unified mathematical model to assess stability. In the present simulations, unfavorable implant mobility indicators contributed to progressive reductions in calculated Implant Stability Index (ISI) values, suggesting increased theoretical implant micromobility and reduced biomechanical stability within the multivariable computational framework. These findings are consistent with previous reports indicating that increased Periotest measurements may reflect impaired implant stability and unfavorable osseointegration behavior [17,18].

Bone density represented one of the major biomechanical variables influencing calculated implant stability within the developed model. Reduced bone density contributed to lower calculated ISI values and an increased theoretical risk of instability. Similar observations have been reported in previous biomechanical and clinical studies demonstrating that poor bone quality may negatively affect primary implant stability, stress dissipation, and long-term peri-implant mechanical support [19,20]. In contrast, increased bone density was theoretically associated with improved biomechanical stress distribution and elevated Theoretical Stability Profile profiles.

Biomechanical loading conditions also demonstrated a substantial influence on the developed computational stability framework. Excessive loading conditions produced progressive reductions in calculated ISI values, particularly when associated with unfavorable systemic or bone-related parameters. Previous finite element and biomechanical investigations have similarly demonstrated that excessive occlusal loading and stress concentration may increase peri-implant strain and contribute to marginal bone loss or implant instability [21,22].

Systemic modifying factors, including smoking, osteoporosis, and diabetes mellitus, were additionally integrated into the model due to their reported influence on bone remodeling and osseointegration processes. Diabetes mellitus has previously been associated with delayed healing, altered bone metabolism, and impaired implant survival rates [23]. Similarly, osteoporosis may negatively influence peri-implant bone density and biomechanical support capacity [24]. Smoking has also been widely recognized as a risk factor for impaired healing and increased implant complications due to its vascular and inflammatory effects on peri-implant tissues [25].

An important aspect of the present study is the development of a theoretical computational risk stratification framework capable of classifying implant stability behavior into low-, moderate-, and high-risk categories. Such computational approaches may contribute to the advancement of conceptual and personalized implant dentistry by facilitating early theoretical identification of patients potentially predisposed to implant instability [26].

From a conceptual perspective, the developed Implant Stability Index (ISI) may serve as a proof-of-concept framework for integrating routinely available biomechanical, clinical, and systemic parameters into a unified computational model for theoretical assessment of implant stability.

Recent advances in digital dentistry, artificial intelligence, and computational bioengineering have increasingly emphasized the importance of conceptual modeling and biomechanical simulation for individualized oral rehabilitation planning [27,28].

The developed Implant Stability Index should be interpreted as a theoretical computational model rather than a clinically validated diagnostic instrument. The present framework was developed using normalized theoretical parameter values and simulated biomechanical conditions intended to evaluate the computational behavior of the developed mathematical system. Consequently, the model does not replace clinical examination or validated implant monitoring methods such as Resonance Frequency Analysis or radiographic assessment [29].

Several limitations should also be acknowledged. The model was developed using theoretical normalized parameters rather than real patient datasets, and the computational simulations were not validated against prospective clinical outcomes.

In addition, the weighting of biomechanical and systemic variables was based on theoretical integration from the previously reported literature rather than on experimentally derived coefficients. Future studies should therefore investigate the clinical validation of the developed Implant Stability Index using prospective implant datasets, finite-element biomechanical analyses, and machine-learning-assisted conceptual approaches [30,31].

An additional limitation of the present framework is that the normalized coefficients assigned to the analyzed variables were introduced for conceptual modeling purposes and were not derived from clinical calibration procedures, regression analyses, or machine-learning training datasets. Consequently, the numerical values should be interpreted as illustrative theoretical coefficients rather than validated clinical weighting factors. Furthermore, the framework assumes that biomechanical, implant-related, and systemic variables contribute independently through a simplified multiplicative structure. In reality, the interactions among smoking, diabetes mellitus, osteoporosis, bone quality, implant geometry, and biomechanical loading are substantially more complex and may involve nonlinear, synergistic, and time-dependent effects that cannot be fully captured by the present model. The framework also does not incorporate temporal variables related to healing dynamics, osseointegration progression, peri-implant tissue adaptation, or long-term biological responses. Future studies should therefore investigate alternative weighting strategies, perform sensitivity analyses, and explore more advanced modeling approaches, including multivariate regression models, Bayesian prediction systems, finite element-assisted analyses, machine-learning techniques, and neural-network frameworks.

Despite these limitations, the present conceptual computational framework provides a proof-of-concept approach for integrating biomechanical, implant-related, and systemic variables into a unified theoretical stability assessment structure. The proposed Implant Stability Index (ISI) should be interpreted as an exploratory and hypothesis-generating framework intended to support future validation studies rather than as a clinically applicable conceptual tool.

## 5. Conclusions

The present study developed a conceptual Implant Stability Index (ISI) framework that integrates biomechanical, implant-related, and systemic variables into a unified computational assessment structure. The proposed framework demonstrated the feasibility of computationally exploring theoretical implant stability behavior under different simulated biomechanical and systemic conditions.

The ISI should be interpreted as a proof-of-concept computational framework rather than a clinically validated tool. Future studies should focus on validation using clinical datasets, finite-element analyses, and advanced computational approaches, including artificial intelligence and machine-learning techniques, to assess its potential clinical applicability in implant risk assessment and treatment planning.

## Data Availability

The original contributions presented in the study are included in the article, further inquiries can be directed to the corresponding author.
